# ‘Memories we treasure’: Evaluating the impact on co-designers and audiences of a photographic exhibition by participants with memory problems

**DOI:** 10.1177/14713012251338551

**Published:** 2025-04-25

**Authors:** Sinéad McIntyre, Claudia Cooper, Natalia Chemas, Sukey Parnell Johnson, Paul Higgs, Wendy Martin, Sarah Morgan-Trimmer, Alexandra Burton, Michaela Poppe, Ellie Whitfield

**Affiliations:** Centre for Psychiatry and Mental Health, Wolfson Institute of Population Health, 4617 Queen Mary University of London, London, UK; Faculty of Medical Humanities, Kings College London, London, UK; Division of Psychiatry, 4919 University College London, London, UK; Department of Health Sciences, Brunel University London, London, UK; Department of Health and Community Sciences, 3286 University of Exeter, England, UK; Centre for Psychiatry and Mental Health, Wolfson Institute of Population Health, 4617 Queen Mary University of London, London, UK; Department of Behavioural Science and Health, 4919 University College London, London, UK; Centre for Psychiatry and Mental Health, Wolfson Institute of Population Health, 4617 Queen Mary University of London, London, UK

**Keywords:** visual methods, audience responses, dementia, mild cognitive impairment, stigma

## Abstract

Visual research, including photovoice methods are increasingly used to elicit the experiences of people living with memory loss, though few such studies have investigated the impact of produced images on audiences. Drawing on Freire’s empowerment pedagogy, we aimed to explore how a photography exhibition, created by individuals with memory concerns participating in the APPLE-Tree (Active Prevention in People at risk of dementia through Lifestyle, bEhaviour change and Technology to build REsiliEnce) dementia prevention programme contributed to an understanding on life with memory loss, and whether it reduced any stigma, among its co-producers and audience. Approximately 200 people attended the exhibition launch, of whom 97 completed a survey. We interviewed two co-producers with lived experience of memory problems and seven academic co-producers. In our thematic analysis of survey responses and interview transcripts, we developed three themes: (1) Increasing understanding by giving voice to participants: seeing unique individuals behind the work; (2) The power of visual methods to communicate research findings: interviewees described how the quality of the works and exhibition created an atmosphere that honoured participants as artists, valuing and respecting their messages; (3) Evoking emotions: fear and hope: The audience described the exhibition’s power to evoke strong emotions of fear at the risk of losing “treasured memories” and hope of living successfully with memory loss. Some attendees and study team members felt motivated to try to reduce their future dementia risk. We consider the value of public engagement using arts-based co-production, and the impact of the exhibition in this field. We explore how our findings reflect notions of empowerment in two juxtaposing ways: empowerment to express how living with memory loss feels as a means of activism to reduce associated stigma, and empowerment to change future dementia risk through lifestyle changes.

## Introduction

Ageing is associated with stigma and misconception, especially within visual representations (Twigg & Martin, 2015). Often, visual depictions of ageing are stereotyped or polarised as a ‘heroes of ageing’ or ‘bodily decline’ binary (Featherstone & Wernick, 1995), with images either ‘nostalgic/melancholic’ or ‘humorously carnivalesque’ (Parnell Johnson, 2018). Such stereotypes can be harmful (Gerbner, 1998), lowering self-esteem in older adult populations (Harwood, 1997). Improving representation, diversity, and authenticity of images can support positive self-identity in older populations (Ylänne, 2015).

### Memory concerns in people without dementia

Subjective Cognitive Decline (SCD) (subjective cognitive concerns in absence of objective deficits) and Mild Cognitive Impairment (MCI) (objective cognitive deficits without significant associated functional impairment) are conditions or experiences that do not meet criteria for a dementia diagnosis but are often associated with ageing. Around one quarter of adults aged 60 and over report SCD (Röhr et al., 2020) and 17% of the UK population aged 65+ are living with MCI (Richardson et al., 2019). Many people living with memory loss experience stigma, which diminishes their confidence (Nguyen & Li, 2020).

Although many people with SCD or MCI never develop dementia, they are at increased risk of doing so. Hallam (Hallam et al., 2022) found that half of individuals who reported memory concerns to their GP received a dementia diagnosis within 3 years. SCD and MCI are risk factors for dementia, rather than diagnoses or illnesses, but they can cause distress, social withdrawal, and a decrease in leisure activities (Parikh et al., 2016). Many who experience them fear developing personality changes, further cognitive loss, losing independence or social skills, or being a burden to others. This fear can lead to a reluctance to learn about personal dementia risk (Rosenberg et al., 2020). Improving understanding and changing attitudes to ageing and cognitive decline in society, and amongst healthcare professionals, could improve the quality of life of people living with memory loss. It could also support dementia prevention by reducing barriers to help-seeking, diminishing social consequences and felt stigma, and increasing the development of supportive interventions (Renn et al., 2021).

### Photovoice in people with memory loss

Photovoice is a participatory method in which individuals observe and photograph their everyday realities, then discuss the meanings behind those images (Baker & Wang, 2006). It is an accessible method often used to involve people excluded from more traditional research methods. Wiersma (Wiersma, 2011) and Genoe and Dupuis (Genoe & Dupuis, 2013) pioneered photovoice with people with dementia, to understand and communicate their experiences of living with memory loss and leisure time respectively. They described the practical challenges of conducting photovoice with this group, but noted the opportunities it afforded to engage them as equal partners in research, and for cuing memory and capturing meaning. In recent years, photovoice has been successfully employed with people with dementia and intellectual disabilities (Watchman et al., 2020), MCI and early dementia, and physical disabilities (Jenkins et al., 2025), and to consider how people with dementia use landmarks when navigating outdoors (Seetharaman et al., 2021). A group in Canada used photovoice to elicit the experiences from Southern Inuit communities around dementia prevention (Pace, 2020); they noted the importance of place and culture in supporting health and resilience.

In one of the key theories shaping photovoice, (Freire, 1978) argues that photographs can act as mirrors to communities; he believed that through collective reflection and discussion of images, communities could uncover and challenge constructs that maintain their marginalisation. Wang and Burris (*Photovoice: Concept, Methodology, and Use for Participatory Needs Assessment -* Wang & Burris, 1997, n. d.) extended Freire’s principles and encouraged participants to engage with those in power to facilitate social change. One means of enabling this is through co-producing exhibitions of work created through photovoice projects. MacDonald et al. (Macdonald et al., 2022) reviewed 30 photovoice research studies that included exhibitions, finding that only two assessed the audience’s responses (Maratos et al., 2016; Schleien et al., 2013) – and responses captured were limited to family and friends of co-researchers. A critical gap was therefore identified in evaluating the impact of such exhibitions. MacDonald explored what audiences learnt, felt, and did (what changed) after viewing self- portraits and stories by women with disabilities. They found that, by engaging with photographs and stories, audiences learnt about the disability experience and believed it would change other people’s views too.

### The APPLE-Tree photography exhibition

We conducted a visual research project involving seventeen older adults experiencing subjective or objective memory loss, receiving a cognitive wellbeing group intervention (the APPLE-Tree programme) designed to prevent cognitive decline and dementia (Poppe et al., 2022). In our study that drew on photovoice methods, participants worked with a photographer/artist (SPJ). We aimed to represent their experiences of memory concerns, involvement in the APPLE-Tree programme, and things that are important to them, through arrangements of photographs and accompanying texts. Creating work for the exhibition titled “Reimagination: The Reframing of Memory”, was a collaborative process, where rapport and familiarity developed between a photographer/artist, participants, and researcher, to explore and communicate sensitive or nuanced concepts (Reyes et al., 2022). See Whitfield (Whitfield et al., 2023)for details of the photograph workshops and the exhibition.

The exhibition aimed to share insight into the lives and experiences of APPLE-Tree participants with the public (Whitfield et al., 2023). It included photographs with descriptions. A video showing the full exhibition is available online: https://www.youtube.com/watch?v=BRmF99h4L9Y. To our knowledge, this is the first project that invited participants experiencing SCD or MCI to engage the public through exhibiting photographic work reflecting their experiences in a photovoice-style study. Rather than focusing specifically on representations of memory concerns and dementia prevention, these were contextualised in participants’ broader lives and experiences, with visual arrangements and accompanying texts reflecting things, people, memories, and connections that are important to them.

Consistent with wider photovoice aims and Freire’s empowerment pedagogy, we aimed to explore whether the APPLE-Tree exhibition could increase understanding and reduce stigma around SCD/MCI among those coproducing and viewing the exhibition. We aimed to explore how a photography exhibition, featuring works created and shared by individuals with memory concerns, contributed to greater understanding of life with memory loss and reduced stigma towards this population, among both its co-producers and audience members.

## Method

### Study design

To capture the exhibition impact, this qualitative study collected and integrated data from two sources: a qualitative survey distributed to all exhibition launch attendees, and individual qualitative interviews with the study team who developed the exhibition, in the weeks after its launch. Qualitative surveys are an underutilized tool, described as providing both a ‘wide-angle lens’ and the potential for rich and focused data, even where individual responses are brief. The opportunity to record quicky the views of a large and unknown population accorded with our aim to capture the exhibition impact. The ‘anonymous’ mode of responding, which can mean participants feel comfortable ‘talking back’ to the researcher reduced the risk of social desirability bias (Braun et al., 2021). While Braun and Clarke refute the belief of some that qualitative surveys must be supplemented with interviews to provide data of adequate depth and richness (Braun et al., 2021), we do triangulate the audience perceptions with those of the co-designers, captured in interviews. While as a study team we could have included these views more informally as a co-author group, we used these more formal methods to address potential power imbalances in the team, ensuring the perceptions of those with lived experience of memory concerns were valued equally with other participants (Lord et al., 2022).

### Ethics approvals

We obtained approval from the London Research Ethics Committee (Reference 19/LO/0260) and UK Health Research Authority study in March 2020, and an amendment dated 16.12.2021 included the coproduction of the exhibition by APPLE-Tree participants. The UCL Research Ethics Committee (Reference: 22837/001) approved the exhibition survey component of the study in July 2022. Surveys were completed anonymously, and we did not collect identifiable information. Survey respondents did not complete consent forms, but were advised, in line with our ethics approval that completing the survey implied consent for data analysis and publication, including quotes from responses, and that no respondents would be identifiable in any publication. Study team members who were interviewed were sent an information sheet prior to the interview and provided written, informed consent to participate.

### Study context

The APPLE-Tree exhibition was co-produced by a team of nine study members, who developed the project and worked with 13 APPLE-Tree participants to produce the exhibition. This team included two Patient Public Involvement (PPI) members with lived experience of caring for people with memory problems, a photographer/artist, and six academic members (Whitfield et al., 2023). The first night of the exhibition was held at the Wellcome Trust in Central London in September 2022. There was a drinks reception, musical entertainment, speeches by celebrities with an interest in memory loss, and a screening of an informational film about the project, in which the study team and participants discussed the exhibition. Around 200 guests attended the free, public event, including local dignitaries, academics, clinicians and members of the public. Participants exhibiting works were invited to attend and invite family and friends.

Individuals were invited through the APPLE-Tree research study, and clinical and academic networks. Members of the public were also welcomed to attend through newsletter adverts from third sector, local authority and arts sector organisations, and via social media.

### Study sample

We invited exhibition launch attendees to complete the questionnaire; and qualitatively interviewed all study team members.

### Procedure

#### Exhibition launch questionnaire

Attendees were invited to complete the survey when entering the exhibition. Participant information sheets on display outlined the purpose of the research and how data would be used. A QR code was displayed, which attendees could scan if they preferred to read the information and complete the survey via Microsoft Forms. The survey, developed by the study team comprised four questions – attendees were asked to self-report age, gender, and answer open-ended questions: “Why did you come to the exhibition today?”, “What struck you most in the exhibition and/or film?” and “What messages will you take away?”. Blank cards were available at the exhibition where attendees were invited to write or draw freely without prompts about their feelings or thoughts towards the exhibition. Attendees posted completed responses in a shielded letterbox for anonymity.

#### Interviews

Following the exhibition, SM conducted semi-structured interviews with all nine study team members on Zoom, using a topic guide (Appendix A). These were recorded and professionally transcribed. The interviewees had varying levels of involvement in the exhibition planning. Most of the study team attended the exhibition, with some seeing it as a completed body of work for the first time. Interviewees did not complete survey questionnaires.

## Data analysis

Survey responses were transcribed into a spreadsheet by SM and NC. Gender and age categories were analysed using descriptive statistics. Responses to the first open-ended question from the survey: “Why did you come to the exhibition today?” were read independently by SM and grouped by reasons, which were described using frequencies. We also used descriptive statistics to report characteristics of interviewees.

We conducted a reflexive thematic analysis (Braun & Clarke, 2019), across two textual data sets - responses to the open-ended survey questions (What struck you most in the exhibition and/or film? What messages will you take away?) and transcribed interviews. Firstly, SM read through all the data and created nodes (using Nvivo) for all data related to the research question. As patterns were identified amongst these topics, SM organised and refined nodes into major themes and sub-themes. The themes and sub-themes were discussed with NC and then EW, who familiarised themselves with the data. Through this discussion, they further developed the themes, which were then discussed with the wider author group. For example, nodes we named as: *individuality*, s*haring experiences* and *empathy, self-reflection* were developed, with discussion into our first theme, as collectively they showed how the individuality of messages, through provoking self-reflection, increased understanding about memory loss. Our second theme, about the power of visual methods was informed by nodes including: *unique research outputs, potential impact* and *professional execution*. We integrated findings from our two datasets- interview and surveys, moving from one dataset to the other to explore similarities and differences, a process previously described as “following a thread” (O’Cathain et al., 2010).

SM is involved in the APPLE-Tree study, but neither SM nor NC were involved in the inception or organisation of the exhibition. Therefore, they evaluated this data from an external role with a relatively distanced perspective. Other co-authors brought insider perspectives: all were involved in the initial study.

## Results

### Demographics

#### Survey

We received 97 responses, all paper questionnaires distributed at the exhibition. One attendee used the blank pages provided to write a haiku responding to the exhibition. Most respondents identified as female (*n* = 63, 65%). The most prevalent age group was 18–34 (*n* = 36, 37%); 22% (*n* = 21) were aged 35–54; 30% (*n* = 29) were aged 55–74; and 11% (*n* = 11) were aged 75+.

We grouped the reasons why respondents reported attending the exhibition into five categories: 14% (*n* = 14) were APPLE-Tree study participants, including exhibitors; 49% (*n* = 48) were invited by an APPLE-Tree participant or study team member; 28% (*n* = 27) attended due to a personal or professional interest; 5% (*n* = 5) worked on the APPLE-Tree study; and 3% (*n* = 3) gave an ‘other’ reason for attending.

#### Interviews

Table 1 shows the demographic breakdown of the 9 interviewees – 7 were female; 2 were individuals with lived experience 6 were researchers, and 1 was a photographer/artist.

**Table 1.** Characteristics of the study team interviewed.

Interviewee	Role	Gender
1	Researcher	F
2	Researcher	F
3	Researcher	M
4	Lived experience	F
5	Researcher	F
6	Photographer/Artist	F
7	Researcher	F
8	Lived experience	M
9	Researcher	F

### Qualitative findings

Across survey and interview data, we developed three themes responding to our research question.

(1) Increasing understanding by giving voice to participants: Seeing unique individuals behind the work
(2) The power of Visual Methods to communicate research findings
(3) Evoking emotions: fear and hope

Our first theme describes how audiences and interviewees valued the exhibition as a respectful space for participants to communicate very individual experiences of memory loss. The distinctive displays and narratives which were viewed as unique while also highlighting shared themes—such as participants’ deep connection to nature and family—deeply moved audiences, evoking empathy for those navigating the challenges of memory loss. Our second theme describes reflections around visual methods as powerful tools, allowing researchers to “delve more deeply” into experiences and for these to be conveyed strongly and accessibly to broad audiences. Our final theme captures the emotions elicited by the exhibition. Viewers expressed feelings of fear, but also many left with messages of hope—recognising that memory loss could be openly discussed, managed successfully with support, and, for some, inspiring motivation to reduce their own risk of dementia.

### Theme 1: Increasing understanding by giving voice to participants: Seeing unique individuals behind the work

The audience and study team appreciated the exhibition as a platform for participants’ experiences. As each piece and story was unique, the exhibition personalised the topic of memory loss, evoking empathy:

‘It’s very important to me that everyone has their voice in all of these things, and it really did feel like that, it felt like it was properly a communal act and really reflective of deep insights into people’s personal journeys and that was very touching.’ (Interviewee 6)

Although many of the displays incorporated common themes, attendees felt that one of the most surprising aspects of the exhibition was the uniqueness of the works - ‘*I was amazed at the diversity of images*’ (survey - 18–34, woman, personal/professional interest).

The respondents praised ‘*the creativity and the individual ideas and lives*’ (survey – 35–54, woman, personal/professional interest), and ‘*the variety of views and perspectives from the participants*’ (survey – 35–54, woman, personal/professional interest).

‘It was so different; each person had a completely different perspective. And I suppose what I would have feared would have happened if somebody came in and talked through with each person how you might present yourself, they might end up with something very similar and they didn’t. So, I think [name] and the team actually did it really well because obviously they talked to each person and the person themselves led on where they were and what they were thinking’. (Interviewee 4)

This gave the audience what felt like a true insight into participants’ lives - ‘*it very much felt like it was the story of the people who were exhibiting*’ (Interviewee 7).

Though each participant focused on their own personal stories, their collective narrative of feeling anchored by the natural world and family when memory let them down was amplified through many personal testaments. Audiences reflected on these common themes across the exhibition, and how they provided a deeper insight into the participant’s lives:

‘I was also struck by themes of nature and family and childhood. Nature perhaps because of its enduring nature through time and memory?’ (survey – 18–34, woman, invited by APPLE-Tree team/participant)

‘there were some really nice themes that, sort of, emerged from people’s accounts and one of the biggest was just the importance of family and friends, of social connections. Obviously we know that it is something we’re very aware of but it just felt like people’s worlds were becoming more focused on the importance of the people in their networks and the people around them’ (Interviewee 1)

For many, this increased insight into the participants’ lives had a strong impact - audience and study team members commented that the exhibition was ‘*thought-provoking*’ and ‘*inspiring*’ (survey – 35–54, woman, invited by APPLE-Tree team/participant), and that there were ‘*many touching pictures and stories*’ (survey – 75+, woman, APPLE-Tree participant/exhibitor). They were ‘*surprised how moving it was’* and ‘*surprised how it came together to tell a story*.’ (Interviewee 7)

This impact for many created space for empathy – prompting reflection upon how experiencing memory loss might feel - ‘it feels like, yes, they’re at a crossroads potentially and I think that brought that home to me really and if I got to that crossroads what would I feel like, what would I do.’ (Interviewee 1) – and to consider how their own friends and family have been affected by memory loss or dementia:

‘(name of exhibitor)’s photo book of memories for his father who died of dementia. My dad is currently suffering with early-onset Alzheimer's, aged 60, and is at the stage where he loves reminiscing, but can't remember details. The photo book reminded me of the memories I have with Dad, but also the memories I won't get to have with him - my wedding, the birth of my first child. Thank you [name of exhibitor] - your exhibit has made me feel emotions I don't think I've ever felt. I am now beginning to mourn whilst also recognising the need to spend time with Dad while I can.’ (Survey – 18–34, man, invited by APPLE-Tree team/participant).

By providing this platform for participants with memory loss to showcase their unique stories and creative styles, audience members and academics are provided a glimpse into their real lived experiences, that they may not otherwise see.

### Theme 2: The power of using visual methods as a way to represent research findings

In our second theme, we explored audience and interviewee responses to photographs and the exhibition as a method to communicate research findings. Their responses described how the quality of the works and the format of the exhibition created an atmosphere that honoured the participants as artists and valued and respected their messages.

Audiences valued the photographs and other works as a ‘*creative way of sharing research findings’* (survey – 35–54, man, personal/professional interest). A researcher interviewee also recognised the value of visual data in providing unique perspectives:

‘So, it’s a different kind of data, I guess, the rationale would be that you can get different insights into a phenomenon because the data is different. You can see sometimes connections or contexts between an item of focus and it’s wider setting or connections between people and space, for example, or people and their history.’ (Interviewee 5)

According to respondents, a key factor in the success of achieving such high-quality works that were able to convey the participants messages, was the involvement of a creative professional (SPJ) in curating the exhibition. Respondents noted that this artistic expertise added a unique dimension to the project, enabling outcomes that would not have been possible without such multidisciplinary collaboration. Researchers’ perspective: ‘I think having the artist there really brought it to a different dimension and really enabled the participants to have a, you know, show their ideas and the ideas and visualisation and memory… around memory – but also something around their identity and I always think when you ask people to do photographs… it portrays something of their identity as well’ (Interviewee 9)

Photographer/artist’s perspective: ‘You learn in the making and that’s actually an incredibly important part of this project was that the workshops (when we were also present), a very important aspect of this was to get people to engage with their photographs physically and to move them about, because they start to make literally different neural connections. The images - by moving them and by putting them into different arrangements - it starts to spark other ideas and that’s part of the creative process.’ (Interviewee 6)

Attendees described how visual imagery enabled them ‘*to see the world through their [participants’] eyes*.’ (Survey – 18–34, woman, worked on APPLE-Tree). The visual materials were also credited as evoking emotional reactions that were more salient, memorable, and thought-provoking than textual data alone:

‘Because in some senses, you know, it’s like if you go into a museum and you see photographs you can kind of like imagine what the life of the people in a museum represented is. But here you actually had lives being in some senses turned into metaphors, being turned into more open-ended expressions and things like that and so it was actually much more thought provoking.’ (Interviewee 3)

This thoughtfulness behind the creativity of the exhibition was successful in its aim of portraying the works in an engaging way. Audience members commented consistently on the quality of the exhibition and exhibits. Viewers were *‘really impressed by the quality of the photographs*’ (survey- 18–34, man, invited by APPLE- Tree team/participant) and found them ‘*visually stunning’* (survey – 35–54, woman, invited by APPLE-Tree team/participant). The audience also expressed an appreciation for the creative, interactive presentations of photographs:

‘I really liked the movable post-it notes on one of the pieces to allow you to interact with the piece’ (survey – 18–34, woman, other)

‘I thought it was really important to have tactile elements and the magnetic board where you could move things around that also enabled her to stand next to it and theatricalise her own work, which I felt was a really important formative act for her. It gave her a sense of herself in a different way and it allowed her to distil certain experiences that had been really painful and that was a very powerful outcome.’ (Interviewee 6)

Besides the creative displays, attendees also valued the stories that were displayed next to the images to provide context, as these helped in bringing the images to life:

‘The back stories to each of the pieces were really insightful and moving’ (survey – 18–34, woman, other)

‘Some very moving life stories to accompany their photographers. So much I shall be taking away with me’ (survey – 55–74, woman, invited by APPLE-Tree team/participant)

Respondents also noted another benefit in using visual methods was that it creates an opportunity to make research more accessible to a wider audience than traditional methods:

‘The other thing, of course, and actually I was aware of, was the ethnic diversity certainly in the people who came to look at it… the idea of APPLE Tree is to reach [diverse] people.’ (Interviewee 4)

Through hosting an event accessible to all, and not just academics or clinical professionals, the exhibition launch was an opportunity for a wide range of attendees to engage with each other and for important discussions to emerge:

‘I think just seeing people from so many different areas of life coming together and having a chat over the photos was really very moving. And the fact that the participants were there showing them to their families… it became a truly coproduced exhibition.’ (Interviewee 7)

Audience members gained another layer of understanding to speak one-on-one with the participants and to hear about experiences directly, which provided an opportunity for reducing stigma:

‘I spoke to one of the exhibiters who described why she had selected particular photographs - she wanted to share her experiences and that in itself was a very powerful way of connecting’ (survey – 55–74, woman, personal/professional interest)

‘The participants loved talking about their art so it gave it that extra dimension, didn’t it? Because it’s an exhibition with people and people, you know, the people create the space and the feeling of the exhibition as well as the art, it all comes together, doesn’t it, really’ (Interviewee 9)

‘It wasn’t just about the photography… people sat and talked about it… the thought behind it and how it came about.’ (Interviewee 8)

### Theme 3: Evoking emotions: fear and hope

The audience highlighted the exhibition’s powerful ability to evoke strong emotions, particularly fear and hope. Many were deeply impacted by the reflections on feelings of fear: ‘*maybe the honesty of the participants about their fears and that they were encouraged and willing to share such personal stories. It wasn’t a surprise, but it was in a way,’*

‘And the theme of the piece was about this up and down, I suppose of her life going into the future as well because one of the photos represents her fear of the memory loss and her fears of the future… her mind often goes back to the past these days because of her fears for the future. So, it was all really relating to that, but she really wanted to communicate to people and she really wanted to put that out there.’ (Interviewee 2)

Despite this, audience members and interviewees also left the exhibition with feelings of hope. One commented on ‘*how positive the accounts were*’ (survey – 18–34, woman, personal/professional interest), another ‘*really loved the uplifting overall tone: loved the sense of control participants had over their memory*’ (survey – 18–34, gender unknown, invited by APPLE-Tree team/participant). For many audience members, hope came from the possibility that memory problems could be shared, and help could be sought

‘That it is ok to talk about getting old, losing your memory as well as losing your way’ (survey – 55–74, unknown gender, personal/professional interest)

‘A declining memory is difficult to deal with but is so much easier when you can share your thoughts and concerns with others’ (survey – 75+, woman, APPLE-Tree participant/exhibitor)

While for others, hope was perceived through symbolism within the photography:

‘The participant with the book, you know, and her writing about the apple blossoms and it’s like blurred memories but there’s rosy apples still to be eaten, I thought that was lovely.’ (Interviewee 2)

As one interviewee described, these contrasting emotional responses also spoke to a theme of uncertainty about the future:

‘There isn’t actually a clear navigation plan and, in fact, nobody really knows what is going on or what is going to happen for them.’ (Interviewee 3)

Hope was conveyed by some attendees and study team members through their new-found motivation to try to reduce their future dementia risk. They took away messages around the ‘*degree to which cognitive impairment can be prevented’* (survey – 18–34, woman, invited by APPLE-Tree team/participant); *‘The importance of a healthy lifestyle and keeping in touch/having positive relationships with those around you’* (survey – 18–34, woman, personal/professional interest) and the importance of perseverance for preserving our cognition - *‘The most important message for me was the thought that you need to 'keep going’ and by doing this you feel better, meet more people and are not so lonely’* (survey – 55–74, unknown gender, invited by APPLE-Tree team/participant).

Poignantly, one audience member reflected on *‘the importance of looking after our memory and to look after the memories we treasure’* (survey – 18–34, woman, invited by APPLE-Tree team/participant), while another reflected that even if there is memory decline, hope still exists in that life can still be lived well:

‘I will take away the ideas of living in such a way that loss of memory can be postponed but also that life can be enriched by taking care to incorporate these practices even if the memory does not improve, life can be enhanced’ (survey – 75+, woman, invited by APPLE-Tree team/participant)

## Discussion

We describe how the APPLE-Tree exhibition influenced co-producers and audiences. They valued the exhibition as a respectful space for participants to communicate very individual experiences of memory loss. Audiences were moved by the exhibition, which engendered empathy, fear and hope. Visual methods provide a powerful tool for communicating the experiences of people living with memory loss accessibly to broad audiences. Audiences and interviewees took away messages of fear and hope, that living with memory loss successfully was possible, but also around possibilities of reducing dementia risk.

Our findings that audiences felt they understood more about, and were moved to empathise with, people with memory loss suggests that the building blocks necessary to reduce stigma were present within the exhibition: seeing participants as individuals, empathising, and putting oneself in their shoes, understanding more clearly the struggles they may face and the positive outlooks they have. Viewing the exhibition led to a feeling of familiarity and reduced feelings of otherness towards people with memory problems. By providing participants with the opportunity to share their experiences through their chosen lens, it gave the audience a fresh and authentic perspective on cognitive decline compared more stereotyped representations in other media (Featherstone & Wernick, 1995).

In the third level of Freire’s empowerment pedagogy, communities reach a deeper understanding of how society and power relationships affect an individual’s situation, enabling them to explore ways in which they can contribute to change through their own actions. However, this change cannot be achieved by individuals alone, with a requirement for community members to work together for meaningful change (Freire, 1978). Notions of empowerment are reflected in two distinct ways in our findings: exhibitors were empowered, by each other and their relationship with the artist and wider team to express how living with memory loss feels and to reject associated stigma. They were recruited as participants of a dementia prevention trial which sought a different type of enablement/empowerment, to reduce their dementia risk by making lifestyle changes. These messages can be uncomfortable, as emphasising the possibility of reducing dementia risk may unintentionally suggest that individuals with memory loss are to blame or responsible for preventing its progression (29). This implication is both stigmatising and inaccurate, as while lifestyle changes can lower dementia risk at a population level, they cannot guarantee prevention for any individual (30). Empowering people with memory loss to reduce dementia risk without increasing anxiety, through responsibilisation, is challenging; audience responses suggested that by emphasising the role of community and support, the exhibition might have been more successful in this than more individualistic interventions (Bird et al., 2021). This is critical, as research indicates that especially in more deprived and minoritised communities, societal-level risk factors for dementia may be more significant than those at the individual-level (Bothongo et al., 2022).

Our study has emphasised the importance of giving research participants, particularly those with memory loss whose voices are not often heard, a platform to express their experiences and perspectives. Offering these opportunities counteracts prevailing stigma and bias surrounding ageing and memory loss. By amplifying the voices of these individuals, we contribute to dismantling age-related stereotypes and misconceptions, thereby promoting a more inclusive and understanding society, and potentially creating a platform where dementia prevention messages can be heard and heeded without stigmatisation.

Our findings echo previous research showing that visual storytelling is a powerful tool for disseminating research findings, fostering empathy, and generating impact. In an age where information overload is common, visuals cut through the noise and convey complex ideas in a more accessible manner. As (Twigg & Martin, 2015) wrote, visual representations are a “powerful force” and shape how our “identities, experiences and everyday lives are mediated, experienced, valued and understood” (p. 95). By presenting data and narratives through visual means, we can bridge the gap between academic research and the public, making the information more relatable and digestible.

The use of visual methods not only ensures that research findings reach a wider demographic but also enhances their retention and understanding. After the official opening, the exhibition was displayed in the Houses of Parliament and the Holy Sepulchre Church in London. Similarly to Reyes (Reyes et al., 2022), who call on “gerontologists to engage with visual methods in qualitative research as an innovative tool for community-engaged research that has potential to advance social justice in gerontology”, our findings suggest researchers can achieve more societal change when they invest in developing skills and collaborating with artists, designers, and communication specialists to effectively convey the nuances of ageing and memory loss research to the public.

When engaging with the public through participants’ imagery and stories, it is important to ensure that their work is honoured and celebrated, through providing a supportive and respectful environment that acknowledges the contributions of participants and researchers alike. This approach is important for sustaining the trust of both current and potential participants and creating a positive outcome of their involvement, thereby facilitating ongoing research in ageing and memory loss. These finding echoes work exploring the impact of a theatre production tackling dementia-related stigma, where putative mechanisms responsible for its powerful impact included: “*the skill of the performance, and the quality of the production*” (Schneider, 2017).

### Limitations

Only half of exhibition attendees completed the survey; exhibition attendees were self-selected as those interested in viewing a photographic exhibition of memory loss, so responses are likely to be more favourable than might be gathered from less self-selecting audiences. The most prevalent age group in survey respondents, 18–34, is also the most prevalent age group in inner London where the exhibition was held, while older age groups (55+) were over-represented in the survey population relative to the local population; this suggests that the exhibition was of interest across a broad age range (London’s Population Age, London Poverty, n.d). We subsequently displayed the exhibition at a church and in the House of Commons, but were unable to collect responses from these exhibitions. While we sought to consider reflexivity during analysis, it was conducted within the APPLE-Tree team, albeit with a study team selected for diverse, external perspectives.

Though we aimed to explore whether the exhibition could increase understanding and reduce stigma around SCD/MCI, we did not measure understanding or perceptions of stigma in attendees before they viewed the exhibition. To do so risked overshadowing the exhibition we were evaluating; an audience survey evaluating a community-based arts and sexual health project in Indonesia, one of the few previous projects to survey audiences of an art exhibition used a similar approach, though they triangulated with later interviews with some audience members (Newland et al., 2021). An evaluation of a theatre production tackling dementia-related stigma also surveyed audiences only after the performance (Schneider, 2017). We asked open survey questions, which did not overtly mention stigma, to avoid being too directive. A recent qualitative interview study exploring the perspectives of health staff with hidden disabilities featured in a hospital art exhibition designed to decrease stigma took a similar approach (Worthington & Grainger, 2025).

The impact of the participant artists engaging with audiences at the exhibition launch may have enhanced its impact; at subsequent exhibitions this was not possible, so we co-created short films so future audiences could hear these voices too; including the virtual tour referenced earlier.

## Conclusion

Holding an exhibition to showcase the creative works of participants with memory loss gave audience and study team members insight into the lives of people living with SCD/MCI, and increased understanding to help counteract stigma around having memory concerns. The themes which emerged from responses to the exhibition emphasise the imperative of empowering research participants, leveraging visual methods for public engagement, and celebrating and honouring the work. These points collectively form a foundation for creating a more inclusive, empathetic, and informed society that addresses the challenges of ageing and memory loss with greater compassion and understanding. As researchers, we can champion these principles in our work and advocacy efforts, driving positive change in the perception and treatment of ageing-related conditions.

## Data Availability

Data is available from the corresponding author on receipt of a reasonable request.*

## Appendix A

Topic guide for study team interviews

Thank you for agreeing to be interviewed today, in your role as part of the study team that planned the APPLE-Tree photographic exhibition. As you know, the interview is being tape- recorded and the transcript will form part of our analysis for a paper looking at responses to the exhibition.

Prompts:

• Firstly, could you tell me a bit about your involvement in the project and what role you played?
• How did you first develop the idea for or become involved in the project to create an exhibition? And is the result similar or different to what you might have imagined at the time?
• What are/were your thoughts and feelings after viewing the exhibition?
• Was there anything in particular that struck you as important, effective or moving? (further prompt – what do you think particularly worked)?
• Are there any pieces in particular that made you want to know more?
• Was there anything that you found surprising about the work displayed in the exhibition, either in viewing it as an attendee, or through the process of planning it?
• Do you feel that viewing the exhibition and film, and your involvement with the project, has changed or enriched your own understanding of the experiences of people with memory concerns, and APPLE-Tree participants at all?
• Is there anything that might have been done differently?
• Are there any other thoughts you would like to add?
